# Our Contemporaries

**Published:** 1916-09

**Authors:** 


### OUR CONTEMPORARIES

---

The Medical Pickwick, established by the late Samuel M. Brickner, is now edited by Julian Walter Brandeis, A. M., M. D., 156 W. 121, N. Y.

---

Our Dumb Animals, Boston, Aug. 1916, denounces, under the title of "Human Vivisection in Michigan—The Betrayal of a Sacred Trust," some investigations by Dr. Udo J. Wile of the University of Michigan. These were reported in the February issue of the Journal of Experimental Medicine under the title "Experimental Syphilis in the Rabbit, Produced by the Brain Substance of the Living Paretic." Credit is given Dr. Edmund A. Christian of the Pontiac State Hospital for the use of six paretics. Using ethyl chloride as a local anesthetic, "The skull was trephined over the frontal convolution at a point about one-half to one inch from the midline and well forward of the course of the middle meningeal artery. By means of a long thin trocar needle connected to a syringe a small cylinder of gray and white matter with some fluid from the ventricle was removed."

So far as we can judge, the first half of the title of denunciation employed by Our Dumb Animals is correct. Unless the story is a fake or deliberate misquotation has been practiced, human vivisection was practiced in the literal sense. As to whether this was a "Betrayal of a Sacred Trust," differences of opinion may well exist, although the issue is clarified by the fact that those charged with the offense are employed and paid by the State of Michigan and that the subjects of experiment were public charges of the State. If, for instance, the patients had been the recipients of private care in a hospital avowedly conducted for purposes of investigation, the subjection to experimentation might be considered a quid pro quo, though not dismissing the general ethical and legal principle that the state may intervene in any contract for the interests of the individual and the public. The issue is further simplified by the fact that the operation can not be claimed to be either therapeutically or diagnostically in the direct interests of the patient. While not one of the first magnitude, necessarily involving considerable risk to the patient and while the condition of the patients was presumably such that nothing, even death, could in a sense be considered to their detriment, the operation was nevertheless, one of serious degree, by no means to be compared with the inconvenience of collecting excreta for examination, or with the practically inappreciable danger and pain connected with ordinary blood examinations.

Hence, the issue is as uncomplicated as might be wished. Does the medical profession or the law, countenance human vivisection? Are insane, or other dependents of the state, even including criminals, legitimate subjects for scientific experiment? It should be noted that, except from the standpoint of fanatics who condemn any kind of exploitation of the lower animals, the issue is not even complicated with that of animal vivisection and this question is waived even by the periodical which stands for the general humane treatment of animals. Simply to avoid dodging an issue, we may be permitted to express the opinion that human vivisection or experimentation for purely scientific purposes is improper under any circumstances, with the common sense qualification implied in the allusion to blood examinations, etc. On the one hand, we feel that the life and health of expert, voluntary subjects, willing to become martyrs to science, is worth more than any particular point which they may be willing to risk their lives to establish. On the other hand, we consider any human life as too sacred to warrant its use or risk for scientific purposes. An obvious ethical corollary is introduced when the patient does not realize that he is being exploited. If, after mature deliberation, which must be largely guided by the medical profession, the state formally and legally holds that the value of scientific research warrants the sacrifice, actual or potential, of any of its members, for the welfare of the great majority, as it now holds for serious crime, military purposes, etc., we might without inconsistence alter this opinion but we do not feel that the ethical principle involved can be set aside by private individuals, however worthy their motives.

### A Prayer for Physicians.

From the pay dispensary; from health insurance; from deadbeats; from the incurable urethritis case; from the neurasthenic who keeps a diary; from the detail man who lectures on the physiology of the stomach; from the birth-control crank and other professional sociologists; from the writers of unneeded textbooks; from drug fiends; from the young married woman who is not strong enough to bear a child; from the laboratory that splits Wassermann fees; from breech cases in primips; from obese pigs who won't diet as directed; from night-calls; from fool legislation; from long-winded papers from new kinds of mouth gags and obstetric forceps; from food cranks who tell us how to live for ten days on a dollar and ten cents; from socialistic health boards; from half-baked specialists; from "practical" nurses; from New Thoughtists; from radium fanatics; from over-zealous Freudians; from the preliminary note on a new cancer treatment; from the seekers after false certificates of illness; from standardization "nuts"; from moribund journals, third-rate journals and freak sex journals—good Lord deliver us? --The Medical Times.

